# Intrinsic Charge-Carrier Transport Limitations in ZnFe_2_O_4_ Revealed by Time-Resolved Microwave Conductivity

**DOI:** 10.1021/acs.jpclett.6c00277

**Published:** 2026-04-06

**Authors:** Rohit Kumar Saini, Kumaraswamy Miriyala, Dmitrii Chernykh, Yotam Engel, Alexander Rashkovskiy, Daniel A. Grave

**Affiliations:** † Department of Materials Engineering, 26732 Ben-Gurion University of the Negev, Beer Sheva 8410500, Israel; ‡ Ilse Katz Institute for Nanoscale Science and Technology, 26732 Ben-Gurion University of the Negev, Beer Sheva 8410500, Israel

## Abstract

ZnFe_2_O_4_ has attracted significant interest as a photoanode material for solar water splitting, yet its photoelectrochemical performance remains inferior to that of well-studied materials such as hematite (α-Fe_2_O_3_). Here, we investigate charge carrier dynamics in epitaxial ZnFe_2_O_4_ thin films using time-resolved microwave conductivity (TRMC). TRMC measurements show that ZnFe_2_O_4_ exhibits a carrier yield–mobility product roughly an order of magnitude lower than that of hematite. Furthermore, the microwave photoconductance action spectrum deviates strongly from the optical absorption spectrum, independent of illumination direction, revealing an excitation-wavelength-dependent, subunity yield of mobile charge carriers. Near-band-edge excitation results in reduced photoconductivity, indicating that a substantial fraction of absorbed photons do not generate mobile charge carriers on nanosecond timescales, accounting for the wavelength-dependent external quantum efficiency (EQE) behavior commonly observed in ZnFe_2_O_4_ photoanodes. Overall, these findings reveal inherent limitations in both carrier yield and transport properties, offering a mechanistic basis for the photoelectrochemical performance of ZnFe_2_O_4_.

## Graphical abstract

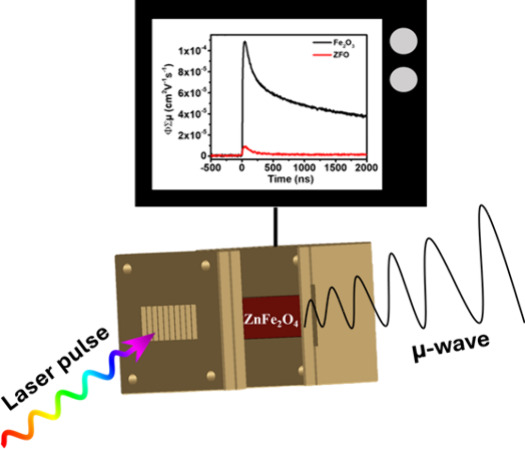

Transition metal oxides have been widely studied as photoelectrode materials for direct photoelectrochemical (PEC) water splitting.^1–4^ In recent years, zinc ferrite (ZnFe_2_O_4_, ZFO) has gained attention as a promising photoanode material due to its bandgap and excellent stability under alkaline oxidative conditions.^5–9^ However, state-of-the-art nanostructured ZnFe_2_O_4_ photoanodes reach saturation photocurrent densities of roughly 1–2 mA/cm^2^ under AM 1.5G illumination, far lower than the theoretical maximum based on its bandgap energy.^7,10,11^ In comparison, champion hematite (α-Fe_2_O_3_) photoelectrodes achieve saturation photocurrent densities of ∼4–6 mA/cm^2^, despite having a similar bandgap.^12–15^ This discrepancy raises the possibility that ZnFe_2_O_4_ is intrinsically limited by inefficient mobile carrier generation and poor transport.

Despite extensive interest in ZnFe_2_O_4_ as a photocatalytic material, experimental insight into its excited-state charge carrier dynamics remains limited. To our knowledge, direct pump–probe studies of ZnFe_2_O_4_ are scarce and are largely restricted to transient absorption measurements on surfactant-capped nanocrystals.^16^ That study reported transient absorption signatures that were interpreted in terms of distinct electronic excitations, including ligand-to-metal charge-transfer (LMCT) and ligand-field (LF, d–d) transitions. However, it remains unclear how excitation into these different optical manifolds relates to the generation of mobile charge carriers that contribute to electrical transport.

Complementary to optical spectroscopies, time-resolved microwave conductivity (TRMC) directly probes the generation and dynamics of mobile charge carriers by monitoring changes in reflected microwave power following pulsed laser excitation.^17–19^ The transient change in conductance induced by the excitation is proportional to the product of the absorptance normalized quantum yield (ϕ) and the combined mobility of the electron and holes (Σμ). Accordingly, the maximum quantum-yield mobility product, ϕΣμ_max_, can be extracted from the signal amplitude and used as a figure of merit to evaluate the semiconducting properties of the material while the decay of the transient microwave signal provides the charge carrier lifetime.

In this work, we apply time-resolved microwave conductivity (TRMC) to epitaxial ZnFe_2_O_4_ thin films grown by pulsed laser deposition. The use of epitaxial films minimizes extrinsic structural disorder, enabling isolation of intrinsic charge-transport behavior. By benchmarking against epitaxial α-Fe_2_O_3_ reference films, we assess whether the underperformance of ZnFe_2_O_4_ photoanodes arises from fundamental limitations in mobile charge-carrier generation and transport. In addition, wavelength-dependent TRMC measurements are used to probe how optical excitation translates into mobile photoconductance.

Initially, ∼400 nm thick epitaxial ZnFe_2_O_4_ thin films were grown on sapphire c-plane (0001) substrates by pulsed laser deposition (PLD) for TRMC characterization. Figure 1 shows high-resolution X-ray diffraction (HR-XRD) analysis of the ZnFe_2_O_4_ epitaxial films including an out-of-plane θ-2θ scan, a rocking curve scan, and off-axis φ scans. All observed reflections in the out-of-plane θ–2θ scan (Figure 1a) correspond to the ZnFe_2_O_4_ spinel phase, with no detectable secondary phases. The presence of only (111)-type reflections confirms a single, well-defined out-of-plane orientation. The rocking curve for ZnFe_2_O_4_ (Figure 1b) exhibited a bimodal profile in the (222) reflection: a sharp central peak with a full width half-maximum (fwhm) of 0.04°, superimposed on a broader component with a width of 1.3°, suggesting the coexistence of highly ordered regions alongside domains with greater mosaic spread.

**Figure 1:**
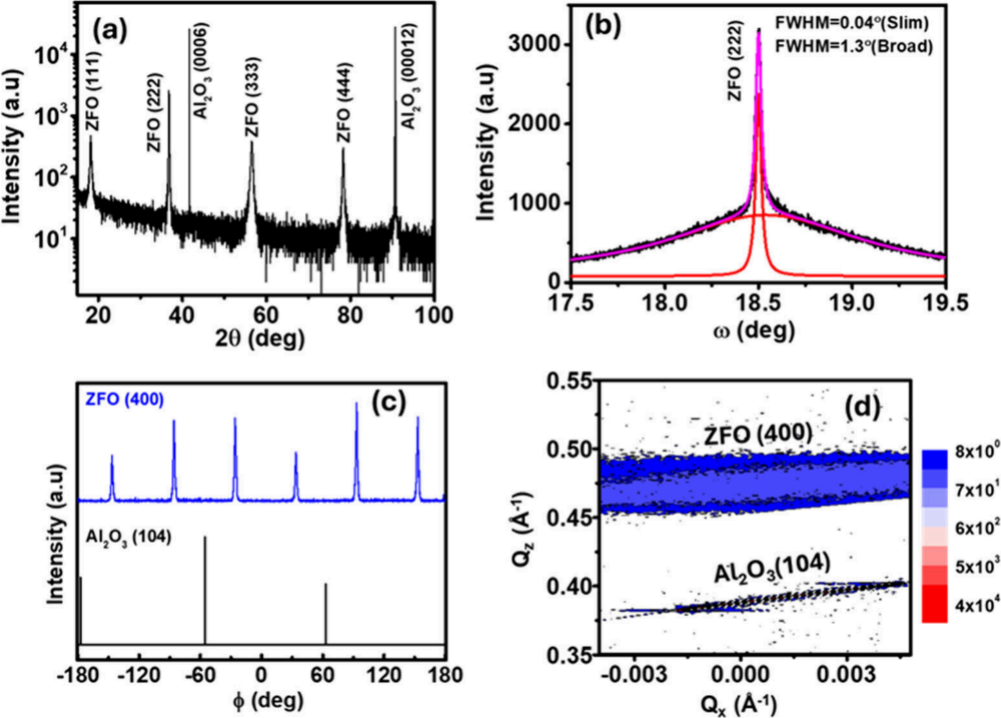
(a) Out-of-plane θ–2θ HR-XRD scan of ZFO thin film. (b) High-resolution rocking curves (RC) of the (222) reflection. (c) φ scan of the (400) reflection. (d) Asymmetric reciprocal space map (RSM) around the ZnFe_2_O_4_ (400) and the sapphire substrate (101̅4) reflections.

The φ scan of the ZnFe_2_O_4_ (400) reflection (Figure 1c) confirms in-plane epitaxial alignment with the underlying sapphire (0001) substrate. Six peaks are observed, separated by 60°, consistent with rotational twinning commonly seen in (111)-oriented spinel ferrite films on hexagonal substrates.^20^ These twin domains likely result from symmetry matching between the 3-fold cubic (111) surface and the 6-fold symmetry of the sapphire (0001) plane. The inclusion of this second in-plane domain could also contribute to the broader component observed in the rocking curve (Figure 1b), reflecting a distribution of misoriented regions within the epitaxial film.

To further confirm the epitaxial nature of the ZnFe_2_O_4_ films, reciprocal space maps (RSMs) were collected around both symmetric and asymmetric reflections. The asymmetric RSM, shown in Figure 1d, was measured around the ZnFe_2_O_4_ (400) reflection and the sapphire (101̅4) substrate peak, revealing broadened film scattering arising from rotational twin variants, while preserving in-plane epitaxial alignment. A symmetric RSM around the (222) reflection (Figure S1) further supports the out-of-plane orientation and crystalline quality of the ZnFe_2_O_4_ films.

To enable a meaningful benchmark, epitaxial hematite films of similar thickness were also grown on sapphire (0001) substrates. HR-XRD shown in Figure S2 reveals only hematite (000l) reflections, confirming a single, well-defined out-of-plane orientation. The hematite film displayed an fwhm of 0.04° in the rocking curve of the (0006) reflection, indicative of minimal mosaic spread and excellent out-of-plane crystalline order. RSMs were also collected for the α-Fe_2_O_3_ reference films and are shown in Figure S3. These data similarly confirm the epitaxial growth and crystalline alignment of the hematite films. The XRD analyses therefore establish high-quality epitaxy in both material systems. Raman spectroscopy further confirms phase purity of both the α-Fe_2_O_3_ and ZnFe_2_O_4_ films (Figure S4), with characteristic vibrational modes consistent with literature reports and no detectable secondary phases. Scanning electron microscopy (Figure S5) reveals a dense, continuous, and crack-free film morphology for both films.

Before examining the TRMC response of the films, ZFO and hematite thin films of identical thickness were grown on conductive Nb-doped SnO_2_ (NTO) coated sapphire substrates to evaluate their photoelectrochemical performance. The epitaxially grown NTO conductive layer enables PEC measurements while maintaining a structural template in the ZFO and hematite films nearly identical to that of the films deposited on the bare sapphire substrate. The XRD patterns of these films are shown in Figure S6, demonstrating that the films grown on NTO-coated sapphire show the same out-of-plane orientation as those grown directly on sapphire.

Linear sweep voltammograms of these films measured under solar simulated light (AM1.5G) in 1 M NaOH solution are shown in Figure S7. The PEC response of ZnFe_2_O_4_ is substantially worse than that of α-Fe_2_O_3_, reaching saturation photocurrents of approximately 100 μA/cm^2^ as compared to 500 μA/cm^2^, respectively. We note the PEC performance is similar to that previously reported for polycrystalline ZFO and hematite thin films grown by atomic layer deposition (ALD) on fluorine-doped tin oxide (FTO) substrates.^8^ These results demonstrate that even in high-quality epitaxial films, ZFO suffers from severe recombination. To isolate bulk transport effects, additional measurements were performed with a hole scavenger added to the electrolyte (1 M NaOH + 0.5 M H_2_O_2_). Under these conditions, surface recombination is suppressed, and the bulk hole flux can be assessed. The measured saturation photocurrents with and without the addition of the hole scavenger for ZnFe_2_O_4_ are similar, confirming that the dominant performance limitations arise from bulk recombination, rather than interfacial kinetics. We note that this underperformance is not limited to the epitaxial planar thin films reported here. Even in nanostructured ZFO photoelectrodes, which are designed to reduce carrier transport distances and enhance surface kinetics, reported photocurrents remain significantly below those of champion α-Fe_2_O_3_ photoelectrodes.^9,21–23^ These deficits across various device designs point toward intrinsic material limitations in ZFO as the dominant factor in its poor PEC activity.

To probe the origin of these limitations, we employ cavity-based time-resolved microwave conductivity (TRMC) as a noncontact method to assess the charge carrier dynamics. By measuring transient changes in microwave conductance following pulsed optical excitation, TRMC provides direct insight into fundamental processes such as charge generation and recombination, while also enabling extraction of key material properties like effective carrier mobility. The relationship between the induced photoconductance Δ*G* and the change in the microwave reflected power $\frac{\Delta P}{P}$ from the TRMC cavity is given by the equation

1 $$\frac{\Delta P\left(t\right)}{P}=-K\Delta G\left(t\right)$$

where K is the sensitivity factor of the cavity, extracted using an impedance model similar to those presented in previous works.^17,25^ The principal figure of merit in TRMC is the product of the quantum yield of carrier generation (ϕ) and the sum of the electron and hole mobilities ∑μ = μ_e_ + μ_h_, expressed as ϕΣμ. This product reflects both the efficiency of mobile charge carrier generation and their ability to move through the material and can be obtained by the relation

2 $$\phi \Sigma \mu =\frac{\Delta G}{I_{0}\beta eF_{A}}$$

where *I*_0_ is the incident photon fluence, β is the ratio between the inner dimensions of the waveguide, *e* is the electron charge, and *F*_*A*_ is the fraction of absorbed photons within the sample. A simplified schematic of the TRMC system used in this work is shown in Figure S8 with a more detailed description provided in the methods section.

TRMC measurements were first performed on the hematite and ZFO films using 350 nm excitation from a laser with a pulse width of approximately 4 ns, under an absorbed photon fluence of 3 × 10^14^ photons cm^–2^ pulse^–1^. The corresponding traces are shown in Figure 2a. α-Fe_2_O_3_ exhibits a peak photoconductivity signal corresponding to a ϕΣμ_max_ of 1.1 × 10^–4^ cm^2^ V^–1^ s^–1^, while ZnFe_2_O_4_ shows a more than order of magnitude lower ϕΣμ_max_ of 9.5 × 10^–6^ cm^2^ V^–1^ s^–1^. This difference in ϕΣμ_max_ highlights the more favorable charge transport properties of hematite under identical excitation conditions. We note the ϕΣμ_max_ value for the undoped α-Fe_2_O_3_ measured here is also in reasonable agreement with previously reported TRMC values for similarly grown epitaxial Sn-doped hematite thin films (∼3 × 10^–4^ cm^2^ V^–1^ s^–1^).^26^ To verify that the measured TRMC signal originates from the film rather than from heating of the sapphire substrate or cavity components, control measurements were performed on a bare sapphire substrate. Because sapphire exhibits negligible absorption at 350 nm, the substrate was excited at an incident photon fluence of 7 × 10^14^ photons cm^–2^ pulse^–1^, corresponding to a higher absorbed fluence than used for the ZFO measurements. Under these conditions, the bare substrate shows a negligible TRMC response (Figure S9), confirming that the observed signals arise from the ZnFe_2_O_4_ and α-Fe_2_O_3_ films.

**Figure 2:**
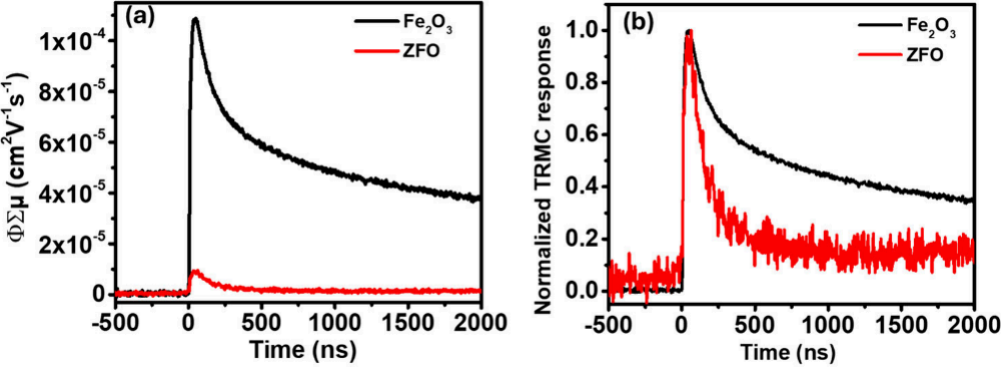
(a) Time-resolved microwave conductivity (TRMC) traces for epitaxial α-Fe_2_O_3_ and ZnFe_2_O_4_ thin films under 350 nm pulsed excitation. Measurements were performed at an absorbed photon fluence of 3 × 10^14^ photons cm^–2^ pulse^–1^. (b) Normalized TRMC transients corresponding to the data shown in (a).

Figure 2b shows the normalized TRMC transients corresponding to the transient photoconductivity data in Figure 2a, allowing direct comparison of carrier lifetimes in ZnFe_2_O_4_ and α-Fe_2_O_3_. The ZnFe_2_O_4_ transient decays substantially faster than that of α-Fe_2_O_3_, indicating more rapid recombination. The combination of the shorter lifetime and lower yield-mobility product in ZFO indicates shorter effective diffusion lengths compared to hematite. To check that the observed decay is not limited by instrumental response, the microwave cavity response time was determined from the resonance spectrum (Figure S10a), yielding a quality factor Q ≈ 800 and a corresponding response time of approximately 31 ns. The instrument response function (IRF) was calculated by convolution of the laser pulse width and cavity response as detailed in the Supporting Information. The analysis shows that the experimental transient clearly deviates from the calculated IRF, confirming that the measured decay is not limited by the cavity response time (Figure S10b).

The dependence of the TRMC signal on absorbed photon fluence was examined for ZnFe_2_O_4_ under 350 nm excitation and is shown in Figure 3a. The extracted ϕΣμ_max_ decreases with increasing fluence, consistent with the onset of higher order recombination processes at elevated carrier densities. At sufficiently low fluences, the recombination dynamics would be expected to be dominated by first order processes, but due to the intrinsically low ϕΣμ_max_ of ZnFe_2_O_4_, a TRMC signal above the noise floor is only observed for absorbed fluences ≥ 3 × 10^14^ photons-cm^–2^-pulse^–1^. As a result, the low-fluence regime in which first-order recombination would be expected cannot be accessed experimentally for this material under the present measurement conditions.

**Figure 3:**
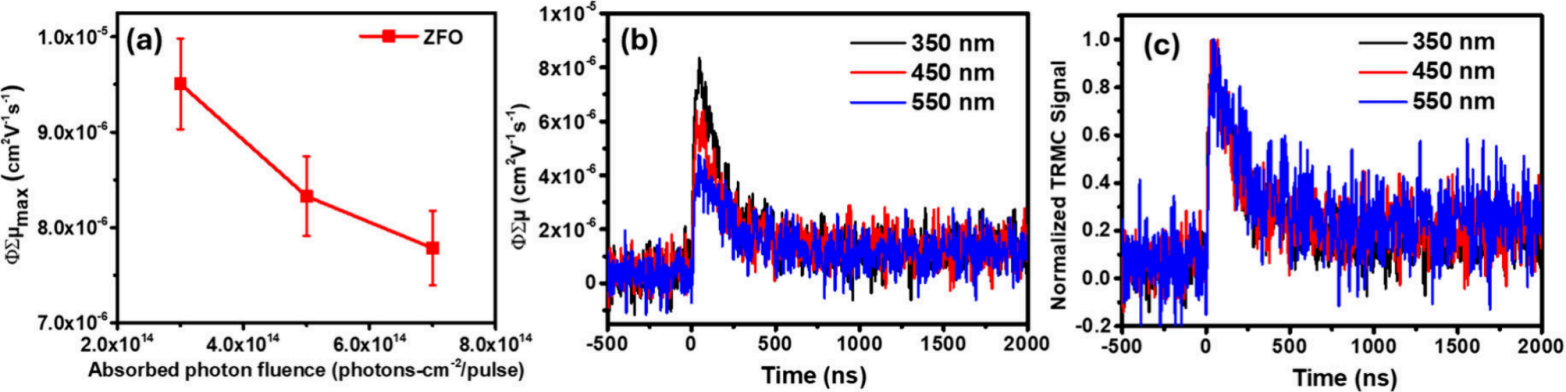
(a) ΦΣμ_max_ extracted from peak of TRMC transients as a function of absorbed photon fluence under 350 nm pulsed excitation. (b) TRMC response measured at various wavelengths under an absorbed fluence of 5 × 10^14^ photons cm^–2^ pulse^–1^. (c) Normalized TRMC response of transients shown in (b).

To evaluate the excitation-wavelength dependence of the TRMC response in ZnFe_2_O_4_, measurements were performed with 350, 450, and 550 nm excitation wavelengths under an absorbed photon fluence of 5 × 10^14^ photons cm^–2^ pulse^–1^. As shown in Figure 3b, the extracted ϕΣμ_max_ depends strongly on excitation wavelength, with the highest value obtained for 350 nm excitation, a modest reduction at 450 nm, and the lowest value observed for 550 nm excitation near the band edge. Since TRMC probes the product of the mobile charge-carrier generation yield (ϕ) and the sum of electron and hole mobilities (Σμ), the observed spectral dependence could, in principle, arise from changes in either quantity. However, when the TRMC transients are normalized by their respective maximum amplitudes, the decay kinetics are identical for all excitation wavelengths (Figure 3b). The identical normalized decay kinetics suggest that Σμ and the dominant recombination pathways likely do not change across the probed wavelengths. Although different optical transitions may be excited at different wavelengths, LF excited states in Fe-based oxides are known to relax on ultrafast, subpicosecond time scales.^27^ Such short-lived states do not contribute to the nanosecond TRMC signal. Consequently, the wavelength dependence of ϕΣμ_max_ is attributed primarily to a reduced yield of mobile charge carriers at longer excitation wavelengths.

For comparison, ϕΣμ_max_ was extracted for α-Fe_2_O_3_ under the same excitation wavelengths and absorbed fluence conditions. At all wavelengths investigated, α-Fe_2_O_3_ consistently exhibits ϕΣμ_max_ values approximately 1 order of magnitude higher than ZnFe_2_O_4_ (Figure S11), demonstrating that the lower photoconductance response of ZnFe_2_O_4_ is not specific to a single excitation wavelength. Reported transport studies indicate that ZnFe_2_O_4_ exhibits extremely low room-temperature mobilities on the order of 10^–8^ cm^2^ V^–1^ s^–1^ as derived from conductivity and Seebeck measurements.^28^ In contrast, reported room-temperature mobilities for hematite under n-type conditions typically fall in the range of approximately 10^–4^ to 10^–2^ cm^2^ V^–1^ s^–1^ depending on defect chemistry and measurement method.^26,29,30^ These literature trends suggest that mobility differences likely contribute to the reduced ϕΣμ_max_ observed in ZnFe_2_O_4_.

To further investigate the wavelength dependence of the TRMC response, measurements were performed at 10 nm intervals from 300 to 600 nm under a fixed incident photon fluence. At each wavelength, the peak photoconductance signal was extracted and normalized by the incident fluence (Δ*G*_max_/I_0_). These values are plotted in Figure 4 alongside the fraction of absorbed photons (F_A_) given by 1 – T – R, where T is the transmittance and R is the reflectance as measured by UV–vis spectrophotometry measurements. Comparative F_A_ spectra for the ZFO and hematite films are shown in Figure S12.

**Figure 4:**
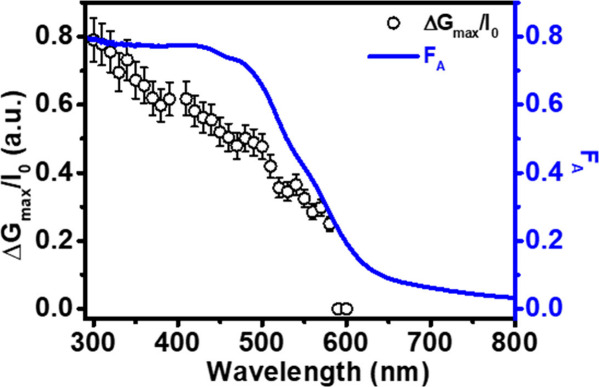
Photoconductance action spectrum of ZnFe_2_O_4_ normalized by incident photon fluence (Δ*G*_max_/I_0_) overlaid with the fraction of absorbed photons, F_A_. Δ*G*_max_/I_0_ was scaled by a factor so both spectra are plotted on the same scale. The measurements were performed under front illumination with a fixed incident fluence of 5 × 10^14^ photons cm^–2^ pulse^–1^.

For a material with unity quantum yield and wavelength-independent mobility, the Δ*G*_max_/I_0_ spectrum at a fixed incident photon fluence would be expected to closely follow the F_A_ spectrum. However, for ZnFe_2_O_4_, Δ*G*_max_/I_0_ exhibits a pronounced maximum at 300 nm and decreases steadily toward longer wavelengths, even though F_A_ remains high until approximately 500 nm. Importantly, this spectral mismatch occurs under both front and back illumination (Figure S13), despite the significant differences in light penetration depth and carrier generation profiles associated with the different illumination conditions. The persistence of this behavior under both illumination directions indicates that the wavelength dependence of the TRMC response is not governed by depth-dependent trapping or surface recombination effects.

Instead, the results suggest that the yield of mobile charge carriers on nanosecond time scales is intrinsically wavelength-dependent in ZnFe_2_O_4_. Longer wavelength excitation appears to generate charge carriers that undergo ultrafast recombination or populate states that do not contribute to photoconductance and therefore are not detected by TRMC. As a result, the common observance of wavelength dependence of the EQE in ZFO photoanodes, where the EQE response decays more sharply than the optical absorption spectrum toward the band edge, can be rationalized by a reduced yield of mobile charge carriers on nanosecond time scales as probed here by TRMC measurements.^31^

This interpretation is consistent with recent photoelectrochemical studies of ultrathin ZFO photoanodes, which report both low charge-carrier yields and short charge transport lengths on the order of 5 nm.^31^ Overall, these findings suggest that carriers generated near the band-edge either recombine rapidly or remain largely immobile, fundamentally limiting charge-collection efficiency in ZnFe_2_O_4_. More broadly, this behavior aligns with recent work showing that metal oxides containing open d-shell cations exhibit ultrafast relaxation via ligand-field states leading to reduced carrier yields compared to metal-oxides based on cations with d^0^ or d^10^ electronic configuration.^27^

In conclusion, we investigated the intrinsic charge transport properties of epitaxial ZnFe_2_O_4_ thin films using TRMC. ZnFe_2_O_4_ exhibits a nearly order-of-magnitude lower yield–mobility product (ϕΣμ) and shorter carrier lifetimes compared to epitaxial hematite, indicating intrinsically less favorable mobile-carrier transport and enhanced recombination. These bulk transport limitations help rationalize the comparatively low charge-collection efficiency and underperformance of ZnFe_2_O_4_ photoanodes. Moreover, the pronounced spectral mismatch between TRMC response and optical absorptance, observed under both front and back illumination, reveals a wavelength-dependent, subunity yield of mobile charge carriers on nanosecond time scales. This behavior provides a mechanistic basis for the commonly observed divergence between optical absorption and external quantum efficiency in ZnFe_2_O_4_ photoanodes.

## Experimental Methods

Epitaxial ZnFe_2_O_4_ (ZFO) and hematite (α-Fe_2_O_3_) thin films were grown on c-plane sapphire (Al_2_O_3_) substrates using pulsed laser deposition (PLD). Dense ZFO and Fe_2_O_3_ targets were prepared using solid state reaction routes from Fe_2_O_3_ and ZnO powders. Commercially available high purity Fe_2_O_3_, ZnO powders (99.99%, Noah Technologies Corporation) were used as initial precursors. To prepare the hematite target, Fe_2_O_3_ powder was ground using a mortar and pestle, then ball milled. Later, the resulting powder was pressed into a 1″ diameter disc pellet and sintered in a box furnace at 1200 °C for 12 h. Similarly, the ZnFe_2_O_4_ target was prepared by grinding the stoichiometrically weighed ZnO and Fe_2_O_3_ powders followed by ball milling the mixture. The resulting powder was pressed into a 1″ diameter pellet and sintered at 1250 °C for 8 h in a box furnace.

Prior to film deposition, the vacuum chamber was pumped down to a base pressure of 5 × 10^–6^ Torr. A KrF excimer laser (Coherent COMPex 102F, λ = 248 nm) was used to ablate the target materials with a laser pulse energy density of ∼1.5 J/cm^2^. For both films, the deposition was carried out at constant set temperature of 800 °C with an oxygen (O_2_) partial pressure of 10 mTorr. During the deposition the laser was operated at a repetition rate of 10 Hz and the target to substrate distance was fixed at 70 mm. After the deposition, the samples were cooled to room temperature under the same O_2_ pressure.

The crystalline structure of the films was examined using a high resolution X-ray diffractometer (Panalytical: Empyrean III) equipped with a Cu radiation source, operated at 45 kV and 40 mA. θ–2θ measurements were performed to obtain the out-of-plane diffraction pattern and rocking curve analysis was used to determine the out-of-plane mosaicity in the films. Off-axis phi scans were employed to identify the in-plane alignment between the film and substrate. Absorptance spectra were obtained from specular reflectance and transmittance measurements using an Agilent CARY5000 UV–vis spectrophotometer.

Raman spectra were collected using a confocal micro-Raman system (Horiba LabRAM HR Evolution) equipped with a Syncerity open-electrode CCD detector. Excitation was provided by a 532 nm laser operated at a power below 32 mW. Measurements were performed using a 100× objective in backscattering geometry with a 600 grooves mm^–1^ grating and a 100 μm confocal pinhole. Spectral acquisition and data processing were carried out using LabSpec 6 software (version 6.8.1.9). Surface morphology was examined using a field-emission scanning electron microscope (Thermo Fisher Verios 460L FE-SEM) operated at an accelerating voltage of 3 kV and a beam current of 50 pA.

Linear sweep voltammograms were performed using a custom-designed electrochemical cell connected to a potentiostat (Palmsens 4) in three electrode configuration. The samples were illuminated under AM 1.5G conditions using AAA solar simulator (Sciencetech). Finally, the LSV curves were carried out in 1 M NaOH alkaline electrolyte solution and in 1 M NaOH+0.5 M H_2_O_2_ hole scavenger solution.

TRMC measurements were performed in a home-built system shown schematically in Figure S8. Tunable wavelength optical excitation was provided by an optical parametric oscillator (OPO) coupled to a flash-lamp pumped Nd:YAG laser (Ekspla NT342C). The intensity of the laser pulse was controlled using a custom-built variable attenuator based on two Rochon polarizers. The laser fluence was measured directly before the measurement using a calibrated energy sensor covered with an identical grating to the microwave cell. An analog microwave signal generator (Keysight EXG N5173B) outputted microwaves in the X-band (8–12 GHz). The signal then passed through a copper waveguide circuit, routing the microwaves toward the cavity cell containing the sample. The sample was mounted at the electric field maximum, 1/4th of the distance along the cavity dimension. The reflected microwave signal was then routed onto a zero bias Schottky barrier diode (Fairview Microwave SMD0218). The resulting voltage signal was then amplified using a Femto HSA-X-1-40 high speed amplifier. An oscilloscope (Keysight EXR104A) triggered by the laser pulse measured the amplified signal. The signal was then averaged over 1000 pulses and the transients recorded.

## Supplementary Material



## Notes

Additional HR-XRD of ZnFe_2_O_4_ and α-Fe_2_O_3_ epitaxial films grown on sapphire and sapphire/NTO, Raman spectroscopy, scanning electron microscopy, TRMC cavity response and IRF calculation, photoelectrochemical characterization, TRMC schematic, and additional TRMC data (PDF)
