# Supplementary material for: Intrinsic Charge-Carrier Transport Limitations in ZnFe2O4 Revealed by Time-Resolved Microwave Conductivity
Source: J Phys Chem Lett. 2026 Apr 6;17(15):4514–9. doi: 10.1021/acs.jpclett.6c00277 (PMC13093659; doi:10.1021/acs.jpclett.6c00277)
Supplement: Supplementary file 1 [file jz6c00277_si_001.pdf]

Intrinsic Charge-Carrier Transport Limitations in $ZnFe_2O_4$ Revealed by Time-Resolved Microwave Conductivity

Rohit Kumar Saini[1+], Kumaraswamy Miriyala[1+], Dmitrii Chernykh[1], Yotam Engel[1], Alexander Rashkovskiy[1], Daniel A Grave[1,2]*

[1]Department of Materials Engineering, Ben-Gurion University of the Negev, Beer Sheva 8410500, Israel
[2]Ilse Katz Institute for Nanoscale Science and Technology, Ben-Gurion University of the Negev, Beer Sheva 8410500, Israel
*Email: dgrave@bgu.ac.il
[+]Equal contribution

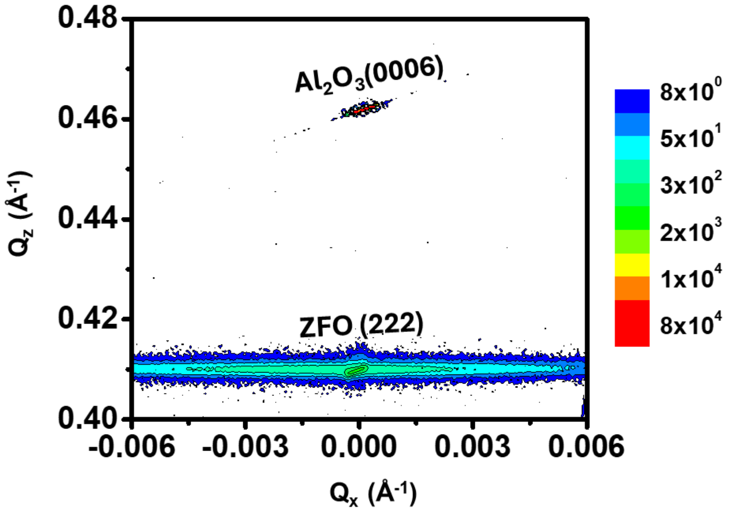

Figure S1. Symmetric reciprocal space map (RSM) around the $ZnFe_2O_4$ (222) reflection.

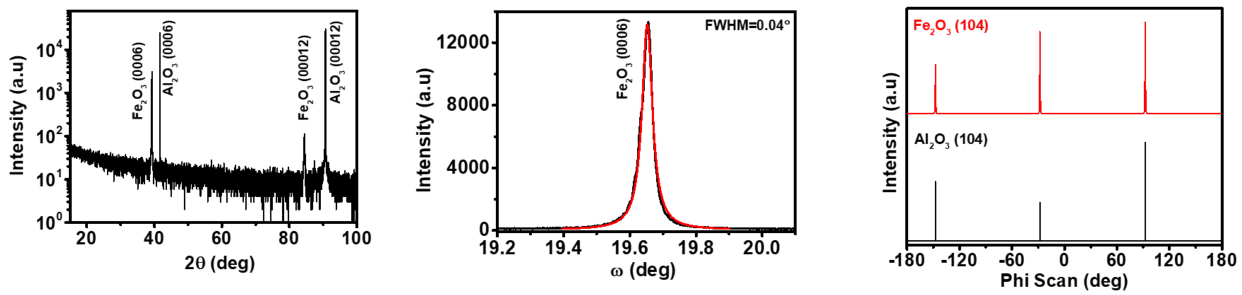

Figure S2. Out-of-plane X-ray diffraction (XRD) θ–2θ scan, rocking curve, and φ scan for epitaxial α-Fe_2O_3 films grown on Al_2O_3 (0001).

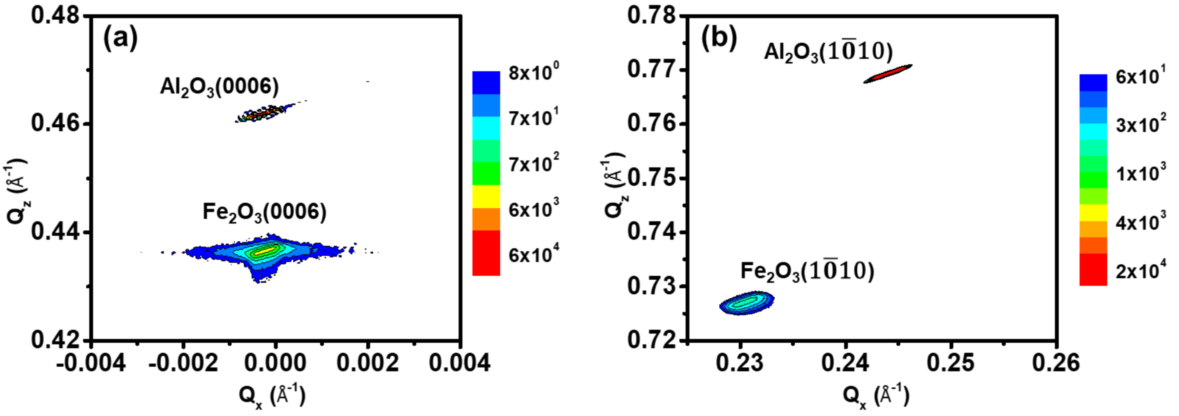

Figure S3. (a) Symmetric RSM around the α-Fe_2O_3 (0006) reflection. (b) Asymmetric RSM around the α-Fe_2O_3 ($10\bar{1}0$) reflection and the Al_2O_3 substrate.

<u>Raman Spectroscopy</u>

Raman spectroscopy was performed to further confirm the phase purity of the Fe_2O_3 and $ZnFe_2O_4$ films. The Raman spectrum of the Fe_2O_3 film (Figure S4a) exhibits characteristic vibrational modes of α-Fe_2O_3, including A_1g modes at approximately 225 and 497 cm^{-1} and E_g modes at 243, 296, 409, and 497 cm^{-1}. An additional infrared-active E_1u(LO) mode is observed near 652 cm^{-1}. These features are in good agreement with previously reported spectra of phase-pure α-Fe_2O_3.[1]

The Raman spectrum of the $ZnFe_2O_4$ film (Figure S4b) displays the characteristic spinel modes, including T_2g modes at approximately 356 and 500 cm^{-1} and an A_1g mode near 672 cm^{-1}, consistent with literature values for phase-pure $ZnFe_2O_4$.[2] No additional peaks corresponding to impurity phases or secondary iron oxide phases were detected in either sample.

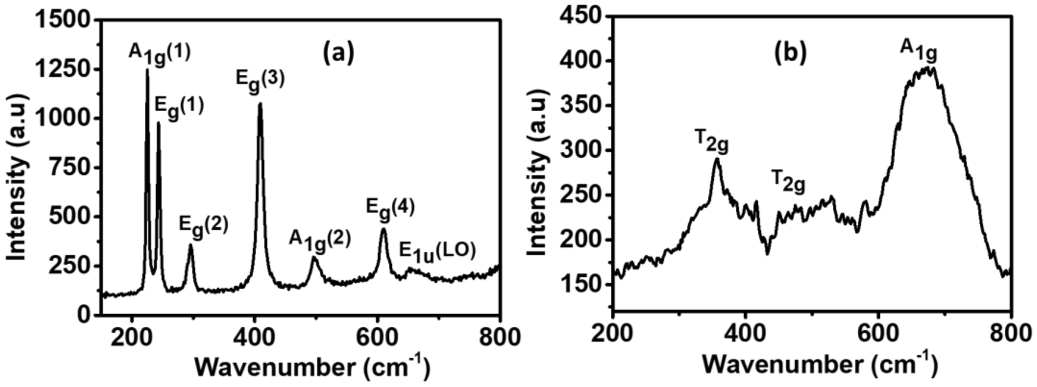

Figure S4. Raman spectra of epitaxial (a) α-Fe_2O_3 and (b) $ZnFe_2O_4$ thin films grown on sapphire substrates.

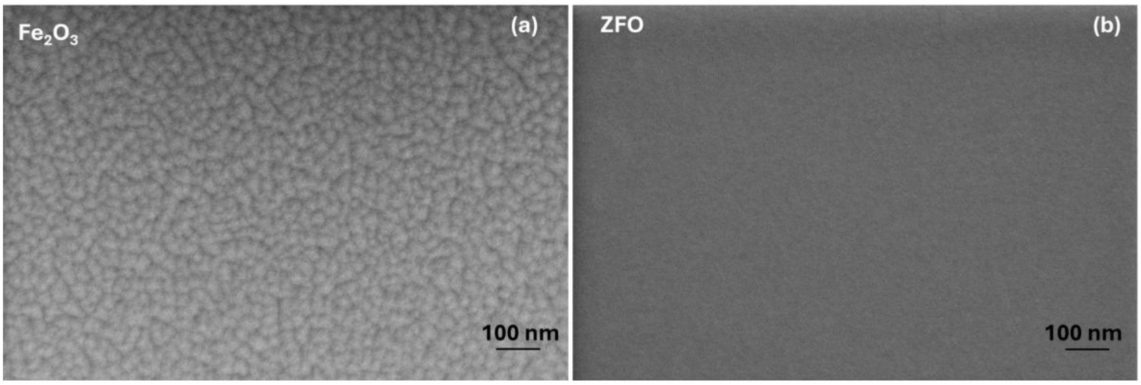

Figure S5. Top-view scanning electron microscopy (SEM) images of (a) α-Fe_2O_3 and (b) $ZnFe_2O_4$ thin films grown on sapphire substrates.

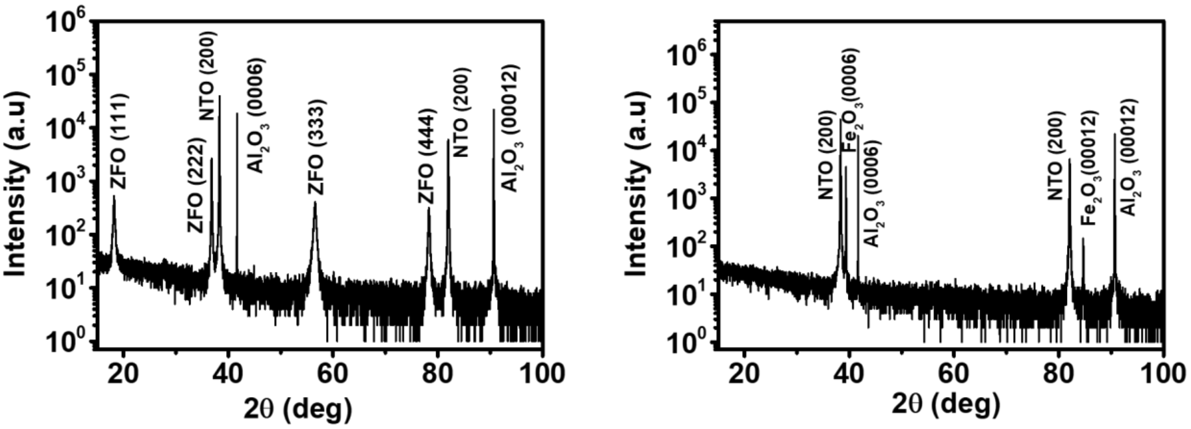

Figure S6. High-resolution XRD patterns of epitaxial $ZnFe_2O_4$ and α-Fe_2O_3 films deposited on Nb-doped SnO_2 (NTO)/sapphire substrates for photoelectrochemical (PEC) measurements.

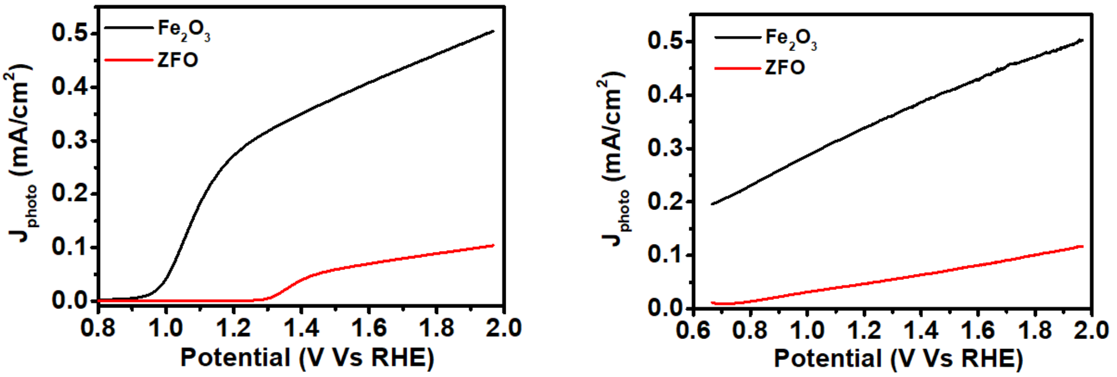

Fig S7. Linear sweep voltammograms of epitaxial $ZnFe_2O_4$ and α-Fe_2O_3 films measured under AM 1.5G illumination in (a) 1 M NaOH solution and (b) 1M NaOH + 0.5 M H_2O_2 hole scavenger solution.

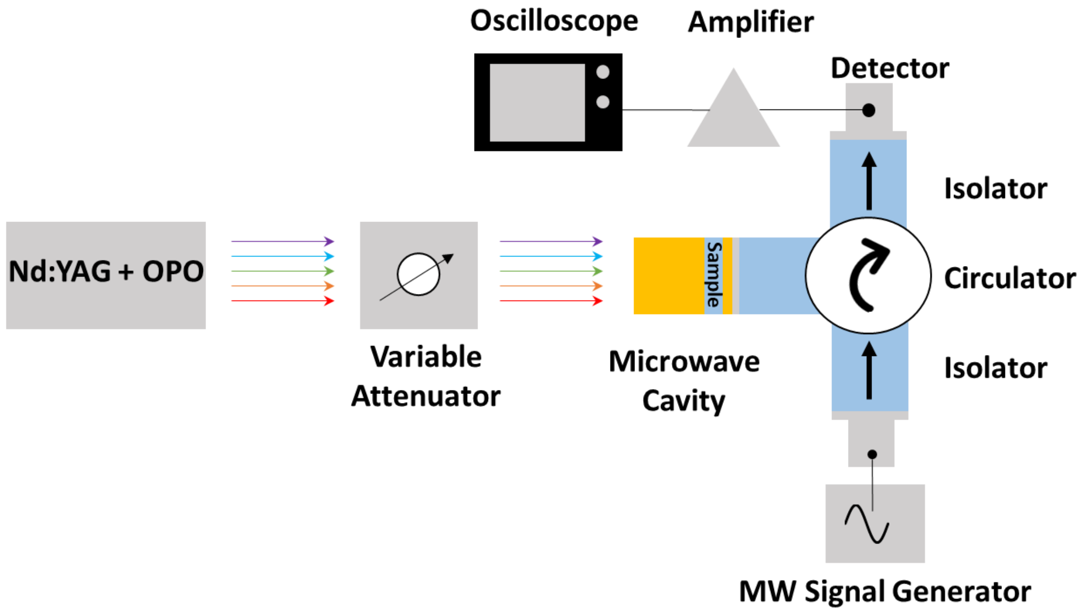

Figure S8. Schematic of TRMC system

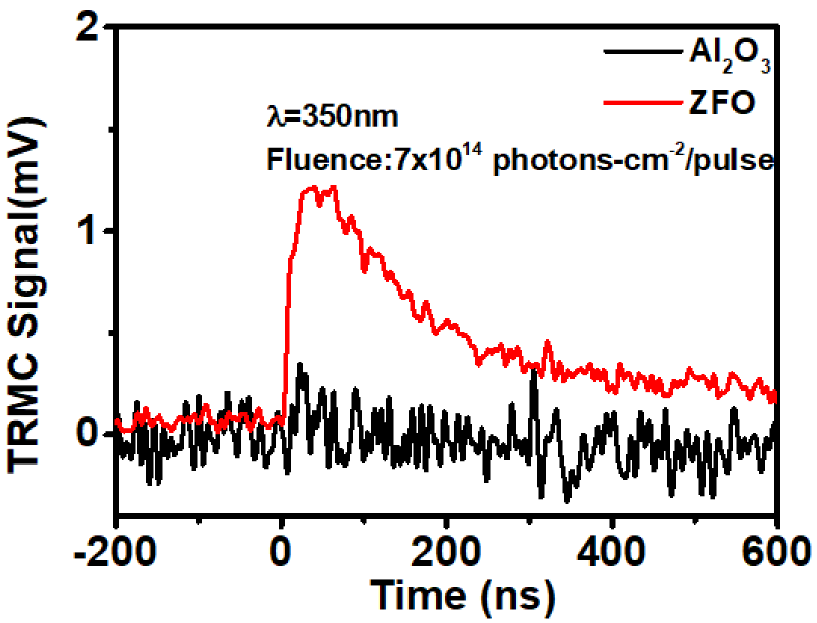

Figure S9: TRMC signal comparison of ZFO film and bare Al_2O_3 substrate at incident photon fluence of 7×10^{14} photons cm^{-2} $pulse^{-1}$

Determination of the Cavity Response Time and Instrument Response Function.

The resonance spectrum of the sample inside the microwave cavity was measured to determine the cavity response time. The resonance curve is shown in Figure S10a. The loaded quality factor was calculated according to $Q_L = \frac{f_0}{\Delta f}$, where f_0 is the resonant frequency, and Δf is the full-width-half-maximum of the resonance peak. The response time of the cavity was then calculated via $\tau_{RC} = \frac{Q}{\pi f_0}$. For the ZFO film on sapphire, the quality factor was found to be 800 and the corresponding response time was 31 ns.

To assess whether the measured transient is limited by the cavity response, the instrument response function (IRF) was calculated following established approaches in TRMC analysis[3] by convolving a Gaussian function representing the laser pulse (rise time ~4 ns) with an exponential decay representing the cavity response. The resulting exponentially modified Gaussian (EMG) function is given by:

$$IRF_{EMG}(t) = \frac{1}{2\tau_{dt}} \exp\left(\frac{\sigma^2}{2\tau_{dt}^2} - \frac{t - t_g}{\tau_{dt}}\right) * Y(t)$$

$$Y(t) = erf\left[\frac{1}{\sqrt{2}}\left(\frac{t}{\sigma} + \frac{\sigma}{\tau_{dt}}\right)\right] + erf\left[\frac{1}{\sqrt{2}}\left(\frac{t - t_g}{\sigma} - \frac{\sigma}{\tau_{dt}}\right)\right]$$

where t_g is the Gaussian centroid, σ is the square root of the variance of the unit-area Gaussian, and $\tau_{dt} = \tau_{RC}$. Using this function, assuming a rise time of 4ns for the laser pulse, and $\tau_{RC} = 31\ ns$ the calculated IRF is overlaid with the TRMC signal from Figure 2b, showing that the measured signal is not limited by the cavity response time.

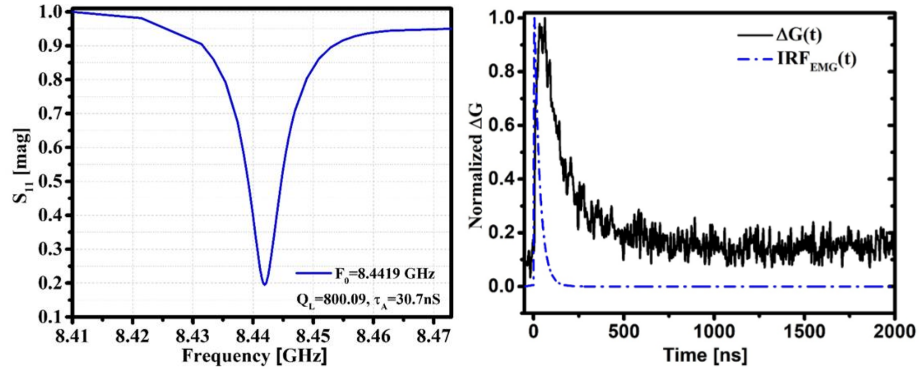

Figure S10 (a). Cavity response of ZFO/sapphire sample (b) Instrument response function (IRF) overlaid with experimental response of ZFO/sapphire sample under 350 nm excitation with an absorbed photon fluence of 3×10^{14} photons cm^{-2}.

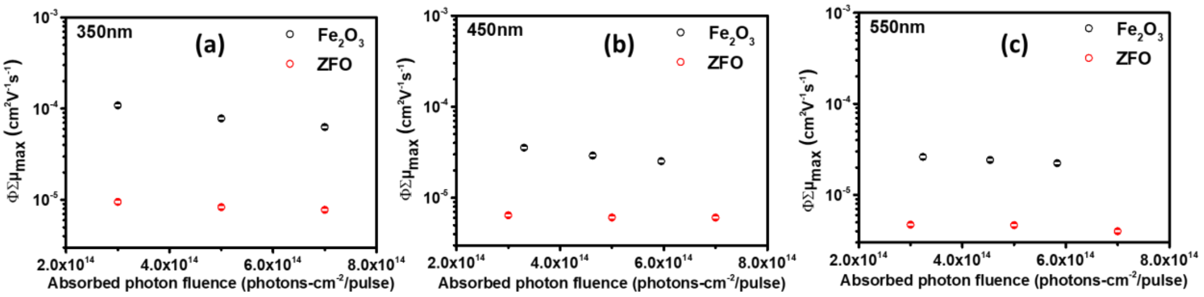

Figure S11. $\Phi\Sigma\mu_{max}$ comparison of ZFO and Fe_2O_3 as a function of absorbed photon fluence at (a) 350, (b) 450 and (c) 550 nm excitation wavelengths.

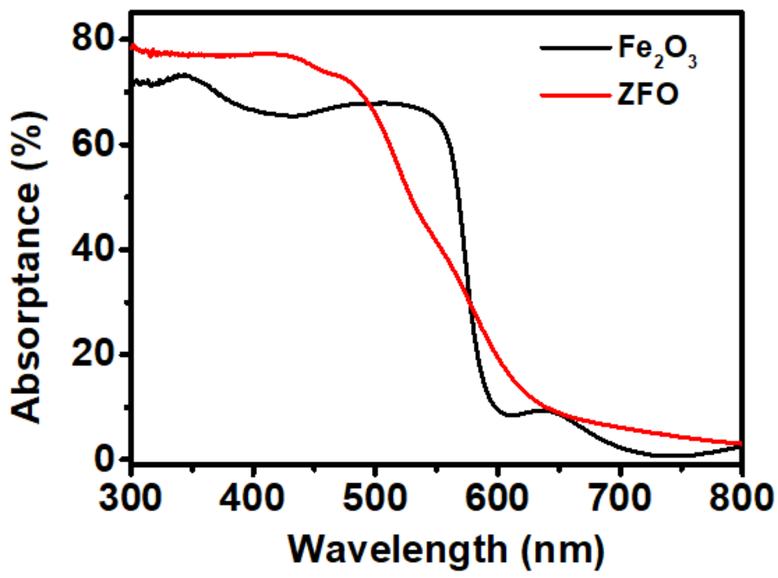

Figure S12. Optical absorptance spectra (A = 1 − T − R) of epitaxial α-Fe_2O_3 and $ZnFe_2O_4$ films deposited on Al_2O_3(0001) measured under front-side side illumination.

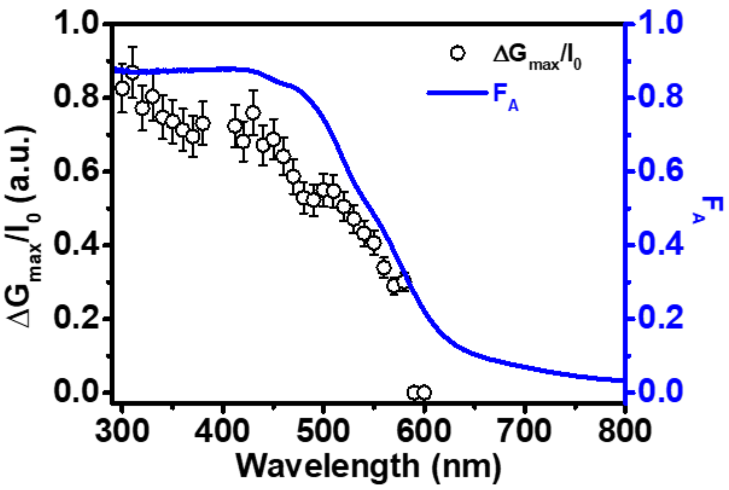

Figure S13. Photoconductance action spectrum of $ZnFe_2O_4$ normalized by incident photon fluence ($\Delta G_{max}/I_0$) overlaid with the fraction of absorbed photons, F_A. $\Delta G_{max}/I_0$ was scaled by a factor so both spectra are plotted on the same scale. The measurements were performed under back illumination with a fixed incident fluence of 5×10^{14} photons cm^{-2} $pulse^{-1}$

References

(1) Pérez León, C.; Kador, L.; Zhange, M.; Müller, A. H. E. In Situ Laser-Induced Formation of α-Fe2O3 from Fe3+ Ions in a Cylindrical Core-Shell Polymer Brush. *Journal of Raman Spectroscopy* **2004**, *35* (2), 165–169. https://doi.org/10.1002/jrs.1125.

(2) Singh, N.; Annadi, A.; Rastogi, A.; Kumar, N.; Sahu, B.; Bhoi, B.; Bohra, M. FMR Study of ZnFe2O4 Thin Films in Varied Growth Environments. *IEEE Trans. Magn.* **2025**, *61* (6). https://doi.org/10.1109/TMAG.2024.3443748.

(3) Savenije, T. J.; Ferguson, A. J.; Kopidakis, N.; Rumbles, G. Revealing the Dynamics of Charge Carriers in Polymer:Fullerene Blends Using Photoinduced Time-Resolved Microwave Conductivity. *Journal of Physical Chemistry C* **2013**, *117* (46), 24085–24103. https://doi.org/10.1021/jp406706u.
